# Endemism patterns in the Italian leaf beetle fauna (Coleoptera, Chrysomelidae)

**DOI:** 10.3897/zookeys.332.5339

**Published:** 2013-09-19

**Authors:** Maurizio Biondi, Fabrizia Urbani, Paola D’Alessandro

**Affiliations:** 1 Department of Health, Life and Environmental Sciences, University of L’Aquila, 67100 Coppito-L’Aquila, Italy

**Keywords:** Coleoptera, Chrysomelidae, Italy, Alps, Apennines, Corsica, Sardinia, Sicily, endemic species, cluster analysis, parsimony analysis of endemicity

## Abstract

In this contribution the results of a zoogeographical analysis, carried out on the 123 endemic leaf beetle species (Coleoptera: Chrysomelidae) occurring in Italy and its immediately adjacent regions, are reported. To assess the level of faunistic similarity among the different geographic regions studied, a cluster analysis was performed, based on the endemic component. This was done by calculating the Baroni Urbani & Buser’s similarity index (BUB). Finally, a parsimony analysis of endemicity (PAE) was used to identify the most important areas of endemism in Italy.

## Introduction

Even if there is not general agreement on whether conservation strategies should focus on hotspots of richness, extinction threat, endemicity or rarity, since the correlation among these factors and their role as biodiversity indicators are still controversial (Bonn et al. 2002; Jetz et al. 2004; Lamoreux et al. 2006; Orme et al. 2005; Prendergast et al. 1993; Reid 1998), the individuation of areas with great endemic species concentration is very important for biogeographical and conservation purposes (Brooks et al. 2006; Burlakova et al. 2011; Whittaker et al. 2005; Wilson et al. 2006). It is important not only for the intrinsic value of the particular species, but also for the funding for projects, since the idea of something exclusive and unique also appeals politicians (Riddle et al. 2011) and lay people (Meuser et al. 2009).

In this contribution, the results of a zoogeographical analysis carried out on the endemic species of leaf beetles (Coleoptera: Chrysomelidae) occurring in Italy, and the immediately adjacent regions, are reported.

Considering the biogeographic purpose of this contribution, we have preferred to follow the “traditional” subdivision into subfamilies as proposed by Lawrence and Newton (1995), and recently also adopted in Löbl and Smetana (2010), for the taxonomy of the Chrysomelidae but Cryptocephalinae and Clytrinae are treated as separated subfamilies. The subfamily Bruchinae was not considered for this study.

Leaf beetles, with 37.000-40.000 described species that are widespread in all the zoogeographical regions, are one of the most species rich families of phytophagous insects (Biondi and D’Alessandro 2012; Jolivet and Verma 2002; Schmitt 1996). In Italy and Corsica there are at least 830 species (D’Alessandro and Biondi 2011; Löbl and Smetana 2010), not including Bruchinae, of which about 15% show varying levels of endemicity.

Distribution patterns in Chrysomelidae are very diverse, varying from cosmopolitan or sub-cosmopolitan species, to species that are strictly and locally endemic. Leaf beetles live in any habitat that has vegetation; because of the species richness of this beetle family and its well documented chorological and ecological information, it is highly representative of the overall biodiversity of a given ecosystem. For these reasons, Chrysomelidae have to be considered an effective instrument for environmental analysis (cf. Hilty and Merenlender 2000). Moreover, this beetle family comprises many species that show high levels of ecological and biological specialization, at least in temperate regions, and a significant trend towards differentiation and endemization in general.

## Materials and methods

In this paper we consider an “endemic species” as “a species showing a distribution restricted to a geographical area, delimited by natural elements, and independent of administrative borders” (cf. Biondi 2006a); whereas an “area of endemism” is “a geographic region comprising the distributions of two or more monophyletic taxa, that exhibit a phylogenetic and distributional congruence, and have their respective relatives occurring in other such defined regions” (cf. Harold and Mooi 1994). Finally, by “subendemic species” we mean “an endemic species not occurring exclusively in a single area”; and “exclusively endemic species”, or “strictly local endemic species”, as “endemic species occurring exclusively in a single area”.

### Study region

**Figure 1.**
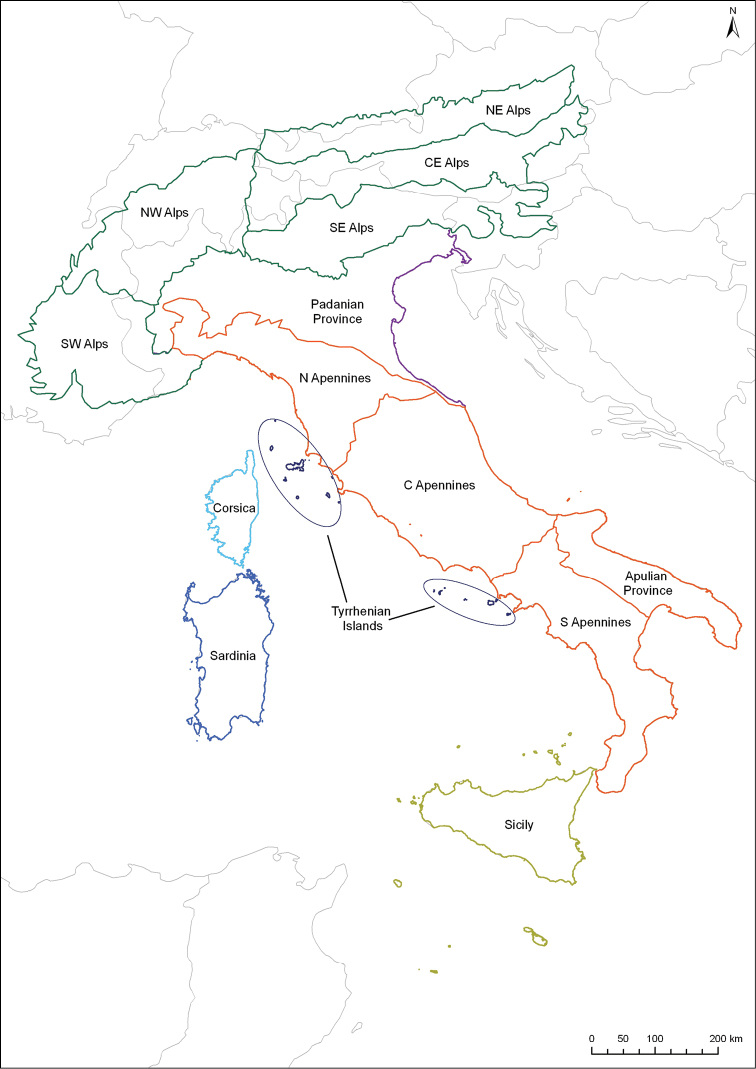
Study region and geographical sectors researched with: CE Alps – Central-Eastern Alps; NE Alps – North-Eastern Alps; NW Alps – North-Western Alps; SE Alps – South-Eastern Alps; and SW Alps – South-Western Alps.

The region studied for this research includes the continental, peninsular and insular parts of Italy, and includes Corsica, as delimited in Fig. 1. The Alps were subdivided into sectors using the SOIUSA method, proposed by Marazzi (2006), which provided the following: the Central-Eastern Alps, North-Eastern Alps, North-Western Alps, South-Eastern Alps, and South-Western Alps (Fig. 1). We refer to the “faunal provinces” proposed by Minelli et al. (2006) for the peninsular and insular parts of Italy, showing the following areas: the Apulian Province, Central Apennines, Northern Apennines, Padanian Province, Sardinia, Sicily, and the Southern Apennines (Fig. 1). Finally, Corsica and the small Tyrrhenian Islands (Capri, Ischia, Pontine Islands, and Tuscany Islands) were added by us (Fig. 1). Distribution maps were constructed by ESRI ArcGis 10.0 software.

### Database

Our database of the endemic species of leaf beetles (Coleoptera: Chrysomelidae), used for the statistical analyses, consisted of: a) records obtained by a critical bibliographic screening (Baviera 2007; Biondi et al. 1994; Biondi 2006c; Kapp 2007; Kippenberg 2008; Leonardi 2007; Moncoutier 2011; Montagna 2011; Sassi 2006, 2011); b) material from entomological collections; and c) information obtained from specialists. The complete list of endemic species, expressed as presence/absence in the different regions studied, is reported in Tab. I. Subspecies were not considered, because their status is often not well defined and also not universally accepted.

The terminology and typology used for the distribution types of endemic and subendemic Italian species follows Vigna Taglianti et al. (1999) and Stoch and Vigna Taglianti (2006), but with the following modifications: a) “3900.22 SISA Sicilian-S-Apennine Endemic” replaces “SISC Sicilian-S-Calabrian Endemic”; b) “3900.23 APSI - Apennine-Sicilian Endemic” was added by us.

Data on host plants were obtained through careful bibliographic screening, integrated with personal observations. Regarding the trophic range, we refer to Biondi (1996), and the following terminology is used:

“monophagous”: species with adults feeding on one or two systematically closely related plant genera;
“oligophagous”: species with adults feeding on plant genera from one or two systematically closely related families; and
“polyphagous”: species with adults feeding on many botanical species that are not closely related systematically.

Finally, the term “herb” refers to species where adults are associated with herbaceous plants, while by “arb/shr” refers to species where adults are associated with trees and/or shrubs.

### Cluster analysis

The level of faunistic similarity between the different geographic regions studied was assessed, based on the endemic component, by performing a cluster analysis which used Operational Geographic Units (OGUs) (see Fig. 1) as reference. This was done by calculating the Baroni Urbani & Buser’s similarity index (BUB) (Baroni Urbani and Buser 1976) on a presence-absence binary matrix of the endemic species. As clustering algorithm, the WPGMA (Weighted Pair-Group Method using Arithmetic averages) was used (McQuitty 1966), and the analysis was done using the MVSP (Kovach 1999) and NTSys (Rohlf 2008) statistical packages. The BUB similarity index is expressed by the formula [(√ad)+a]/[(√ad)+a+b+c], where: *a* is the total number of co-presences; *b* is the total number of species present in OGU 1 but absent in OGU 2; *c* is the total number of species present in OGU 2 but absent in OGU 1; *d* is a total number of co-absences. This index was preferred because we regard the knowledge of the distribution of the endemic leaf beetle fauna in the study area to be good. Thus, we think that co-absences in this analysis represent a highly informative element (Biondi 1988a, 2006b).

### Parsimony analysis of endemicity

Parsimony analysis of endemicity (PAE) was used to identify the most important areas of endemism in Italy (Morrone 1994; Rosen 1988; Rosen and Smith 1988). To increase the reliability of the analysis a selection was made from the data matrix, including only those species that have been reported to live in two or more contiguous OGUs. As geographic reference we used a grid of 50×50 km quadrats superimposed on our study region. Species occurring in only one, or in non-contiguous OGUs, were excluded from this analysis as they are uninformative. PAE was implemented using the MIX programme (1000 randomization, seed = 25) in the PHYLYP package (Felsenstein 1989), with the Camin–Sokal optimization technique (Camin and Sokal 1965), allowing for extinction but not the possibility of new colonization events. For the strict consensus tree (Margush and McMorris 1981; Sokal and Rohlf 1981) the CONSENSE program in the PHYLYP package (Felsenstein 1989) was used.

*Abbreviations*. Distribution types - ALAP: Alpine-Apennine; ALPE: Eastern Alpine; ALPS: Southern Alpine; ALPW: Western Alpine; ALSE: South-Eastern Alpine; ALSW: South-Western Alpine; APPC: Central Apennine; APPE: Apennine; APPN: Northern Apennine; APPS: Southern Apennine; APSI: Apennine-Sicilian; AWNA: Western Alpine-Northern Apennine; CORS: Corsican; ITAL: Italian; PADA: Padanian; SACO: Sardinian-Corsican; SARD: Sardinian; SICI: Sicilian; SISA: Sicilian-Southern Apennine; TYRR: Tyrrhenian.

Geographical sectors - APUL: Apulian Province; CAPE: Central Apennines; CEALP: Central-Eastern Alps; COR: Corsica; NAPE: Northern Apennines; NEALP: North-Eastern Alps; NWALP: North-Western Alps; PAD: Padanian Province; SAPE: Southern Apennines; SAR: Sardinia; SEALP: South-Eastern Alps; SIC: Sicily; SWALP: South-Western Alps; and STI: small Tyrrhenian Islands.

## Results

We found 123 endemic species of Chrysomelidae occurring in the study region (Table 1), which represent about 15% of the total leaf beetle fauna in Italy. This percentage is very high if compared to the total percentage for the endemic component of the terrestrial and inland water fauna in Italy, which is 10%. Seen in the European context, the latter percentage represents a high value (Stoch 2000, 2008).

**Table 1.** Distribution of the endemic species of Chrysomelidae in the geographical sectors studied (for abbreviations see the text).

**Subfamily**	**Species**	**NEALP**	**CEALP**	**SEALP**	**NWALP**	**SWALP**	**PAD**	**NAPE**	**CAPE**	**SAPE**	**APUL**	**SIC**	**SARD**	**CORS**	**TYRR**	**DISTR**
Alticinae	*Aphthona alcina* Leonardi	0	0	0	0	0	0	0	0	0	0	0	1	1	0	SACO
Alticinae	*Aphthona juliana* Springer	0	0	1	0	0	0	0	0	0	0	0	0	0	0	ALPS
Alticinae	*Aphthona perrisi* Allard	0	0	0	0	0	0	0	0	0	0	0	1	1	1	TYRR
Alticinae	*Aphthona sardea* Allard	0	0	0	0	0	0	0	0	0	0	0	1	0	0	SARD
Alticinae	*Aphthona wagneri* Heikertinger	0	0	0	0	0	0	0	0	0	0	0	0	1	1	TYRR
Alticinae	*Dibolia alpestris* Mohr	1	0	0	0	0	0	0	1	0	0	0	0	0	0	ALAP
Alticinae	*Longitarsus bonnairei* (Allard)	0	0	0	0	0	0	0	0	0	0	0	1	1	0	SACO
Alticinae	*Longitarsus gruevi* Leonardi & Mohr	0	0	0	1	1	0	1	0	0	0	0	0	0	0	ALPW
Alticinae	*Longitarsus laureolae* Biondi	0	0	0	0	0	0	0	0	1	0	1	0	0	0	SISA
Alticinae	*Longitarsus nebulosus* (Allard)	0	0	0	0	0	0	0	0	0	0	0	1	1	0	SACO
Alticinae	*Longitarsus refugiensis* Leonardi & Mohr	0	0	1	1	1	0	0	0	0	0	0	0	0	0	ALPS
Alticinae	*Longitarsus springeri* Leonardi	0	0	0	0	0	0	0	1	0	0	0	0	0	0	APPC
Alticinae	*Longitarsus zangherii* Warchalowski	0	0	0	0	0	0	1	1	0	0	0	0	0	0	APPE
Alticinae	*Minota alpina* Biondi	0	1	1	1	0	0	0	0	0	0	0	0	0	0	ALPS
Alticinae	*Neocrepidodera adelinae* (Binaghi)	0	0	0	1	0	0	0	0	0	0	0	0	0	0	ALPW
Alticinae	*Neocrepidodera basalis* (K. Daniel)	0	0	0	1	1	0	0	0	0	0	0	0	0	0	ALPW
Alticinae	*Neocrepidodera ligurica* (J. Daniel)	0	0	0	1	1	0	0	0	0	0	0	0	0	0	ALPW
Alticinae	*Neocrepidodera nobilis* (J. Daniel)	0	0	0	1	1	0	0	0	0	0	0	0	0	0	ALPW
Alticinae	*Neocrepidodera obirensis* (Ganglbauer)	0	0	1	0	0	0	0	0	0	0	0	0	0	0	ALSE
Alticinae	*Neocrepidodera simplicipes* (Kutschera)	0	1	0	0	0	0	0	0	0	0	0	0	0	0	ALPE
Alticinae	*Neocrepidodera spectabilis* (J. Daniel)	0	0	0	1	0	0	0	0	0	0	0	0	0	0	ALPW
Alticinae	*Orestia apennina* Weise	0	0	0	0	0	1	1	1	1	0	0	0	0	0	APPE
Alticinae	*Orestia brandstetteri* Kapp	0	0	0	0	0	0	0	1	0	0	0	0	0	0	APPC
Alticinae	*Orestia carnica* Leonardi	0	0	1	0	0	0	0	0	0	0	0	0	0	0	ALSE
Alticinae	*Orestia carniolica* Weise	0	0	1	0	0	0	0	0	0	0	0	0	0	0	ALSE
Alticinae	*Orestia coiffaiti* Doguet	0	0	0	0	0	0	0	0	0	0	0	0	1	0	CORS
Alticinae	*Orestia electra* Gredler	0	1	1	1	0	0	0	0	0	0	0	0	0	0	ALPS
Alticinae	*Orestia heikertingeri* Leonardi	0	0	1	1	0	0	0	0	0	0	0	0	0	0	ALPS
Alticinae	*Phyllotreta ziegleri* Lohse	1	1	0	0	0	0	0	0	0	0	0	0	0	0	ALPE
Alticinae	*Psylliodes biondii* Leonardi	0	0	0	0	0	0	0	1	0	0	0	0	0	0	APPC
Alticinae	*Psylliodes caneparii* Leonardi	0	0	0	0	0	0	0	0	1	0	1	0	0	0	SISA
Alticinae	*Psylliodes feroniae* Leonardi	0	0	0	0	0	0	0	1	1	1	0	0	0	0	APPE
Alticinae	*Psylliodes fiorellae* Leonardi	0	0	0	0	0	0	1	0	0	0	0	0	0	0	APPN
Alticinae	*Psylliodes leonhardi* Heikertinger	0	0	0	0	0	0	1	0	1	0	1	0	0	0	APSI
Alticinae	*Psylliodes parodii* Leonardi	0	0	0	0	0	0	1	1	0	0	0	0	0	0	APPE
Alticinae	*Psylliodes ruffoi* Leonardi	0	0	0	0	0	0	1	1	1	0	1	0	0	0	APSI
Alticinae	*Psylliodes solarii* Leonardi	0	0	0	0	1	0	1	0	0	0	0	0	0	0	AWNA
Alticinae	*Psylliodes springeri* Leonardi	0	0	0	0	0	0	0	1	0	0	0	0	0	0	APPC
Chrysomelinae	*Chrysolina bourdonnei* Daccordi & Ruffo	0	0	0	0	0	0	0	1	1	0	0	0	0	0	APPE
Chrysomelinae	*Chrysolina osellai* Daccordi & Ruffo	0	0	0	0	0	0	1	0	0	0	0	0	0	0	APPN
Chrysomelinae	*Chrysolina platypoda* Bechyné	0	0	0	0	1	0	0	0	0	0	0	0	0	0	ALSW
Chrysomelinae	*Chrysolina schatzmayri* (G. Müller)	0	0	0	0	0	1	0	0	0	0	0	0	0	0	PADA
Chrysomelinae	*Chrysolina sirentensis* (Meier)	0	0	0	0	0	0	0	1	1	1	0	0	0	0	APPE
Chrysomelinae	*Chrysolina stachydis* (Gené)	0	0	0	0	0	0	0	0	0	0	0	1	1	0	SACO
Chrysomelinae	*Chrysolina suffriani* (Fairmaire)	0	0	0	0	0	0	0	0	0	0	0	1	1	0	SACO
Chrysomelinae	*Chrysolina variolosa* (Petagna)	0	0	0	0	0	0	0	0	1	0	1	0	0	0	SISA
Chrysomelinae	*Gonioctena gobanzi* (Reitter)	0	1	1	0	0	0	0	0	0	0	0	0	0	0	ALPE
Chrysomelinae	*Gonioctena holdhausi* (Leeder)	1	1	1	1	0	0	0	0	0	0	0	0	0	0	ALPS
Chrysomelinae	*Gonioctena lineata* (Gené)	0	0	0	0	0	0	0	0	0	0	0	1	1	0	SACO
Chrysomelinae	*Gonioctena theae* Baviera	0	0	0	0	0	0	0	0	0	0	1	0	0	0	SICI
Chrysomelinae	*Hydrothassa suffriani* (Küster)	0	0	0	0	0	0	0	0	0	0	0	1	1	0	SACO
Chrysomelinae	*Oreina canavesei* Bontems	0	0	0	1	0	0	0	0	0	0	0	0	0	0	ALPW
Chrysomelinae	*Oreina collucens* (K. Daniel)	0	0	0	1	1	0	0	0	0	0	0	0	0	0	ALPW
Chrysomelinae	*Oreina elongata* (Suffrian)	1	1	1	1	1	0	1	1	0	0	0	0	0	0	ALAP
Chrysomelinae	*Oreina genei* (Suffrian)	0	0	0	1	1	0	1	0	0	0	0	0	0	0	AWNA
Chrysomelinae	*Oreina liturata* (Scopoli)	0	0	1	0	0	0	0	0	0	0	0	0	0	0	ALSE
Chrysomelinae	*Oreina melancholica* (Heer)	1	1	1	1	1	0	0	0	0	0	0	0	0	0	ALPS
Chrysomelinae	*Oreina peirolerii* (Bassi)	0	0	0	1	1	0	0	0	0	0	0	0	0	0	ALPW
Chrysomelinae	*Oreina sybilla* (Binaghi)	0	0	0	0	0	0	0	1	0	0	0	0	0	0	APPC
Chrysomelinae	*Timarcha apuana* Daccordi & Ruffo	0	0	0	0	0	0	1	0	0	0	0	0	0	0	APPN
Chrysomelinae	*Timarcha cornuta* Bechyné	0	0	0	0	0	0	0	0	0	0	0	0	1	0	CORS
Chrysomelinae	*Timarcha fracassii* Meier	0	0	0	0	0	0	0	1	0	0	0	0	0	0	APPC
Chrysomelinae	*Timarcha pimelioides* Herrich-Schaeffer	0	0	0	0	0	0	0	0	1	1	1	0	0	1	TYRR
Chrysomelinae	*Timarcha sardea* Villa & Villa	0	0	0	0	0	0	0	0	0	0	0	1	1	0	SACO
Chrysomelinae	*Timarcha sicelidis* Reiche	0	0	0	0	0	0	0	0	0	0	1	0	0	0	SICI
Clytrinae	*Coptocephala raffrayi* (Desbrochers des Loges)	0	0	0	0	0	0	0	0	0	0	0	1	1	0	SACO
Clytrinae	*Labidostomis centromaculata* Gené	0	0	0	0	0	0	0	0	0	0	0	1	1	0	SACO
Clytrinae	*Labidostomis syriaca* Lacordaire	0	0	0	0	0	0	0	0	0	0	0	0	1	0	CORS
Clytrinae	*Lachnaia caprai* Grasso	0	0	0	0	0	0	0	0	0	0	1	0	0	0	SICI
Clytrinae	*Smaragdina ferulae* Gené	0	0	0	0	0	0	0	0	0	0	0	1	1	0	SACO
Criocerinae	*Oulema magistrettiorum* (Ruffo)	0	0	0	0	0	0	1	1	1	0	0	0	0	0	APPE
Cryptocephalinae	*Cryptocephalus albolineatus* Suffrian	1	1	1	1	1	0	0	0	0	0	0	0	0	0	ALPS
Cryptocephalinae	*Cryptocephalus alnicola* Costa	0	0	0	0	0	0	0	0	0	0	0	1	0	0	SARD
Cryptocephalinae	*Cryptocephalus atrifrons* Abeille	0	0	0	0	1	0	0	0	0	0	0	0	0	0	ALSW
Cryptocephalinae	*Cryptocephalus barii* Burlini	0	0	1	0	0	0	0	0	0	0	0	0	0	0	ALSE
Cryptocephalinae	*Cryptocephalus biondii* Sassi & Regalin	0	0	0	0	0	0	0	0	0	0	0	1	1	1	TYRR
Cryptocephalinae	*Cryptocephalus cognatus* Costa	0	0	0	0	0	0	0	0	0	0	0	1	1	0	SACO
Cryptocephalinae	*Cryptocephalus czwalinae* Weise	0	0	0	0	0	0	1	1	1	1	0	0	0	0	APPE
Cryptocephalinae	*Cryptocephalus daccordii* Biondi	0	0	0	0	0	0	0	0	1	0	0	0	0	0	APPS
Cryptocephalinae	*Cryptocephalus equiseti* Costa	0	0	0	0	0	0	0	0	0	0	0	1	1	0	SACO
Cryptocephalinae	*Cryptocephalus eridani* Sassi	0	1	1	1	1	1	1	0	0	0	0	0	0	0	ALAP
Cryptocephalinae	*Cryptocephalus etruscus* Suffrian	0	0	0	0	0	1	1	1	1	1	0	0	0	1	ITAL
Cryptocephalinae	*Cryptocephalus falzonii* Burlini	0	0	0	0	0	0	0	0	1	0	1	0	0	0	SISA
Cryptocephalinae	*Cryptocephalus grohmannii* Suffrian	0	0	0	0	0	0	0	0	1	0	1	0	0	0	SISA
Cryptocephalinae	*Cryptocephalus hennigi* Sassi	0	0	0	0	1	0	1	0	0	0	0	0	0	0	AWNA
Cryptocephalinae	*Cryptocephalus informis* Suffrian	0	0	0	1	1	1	1	1	0	0	0	0	0	0	ALAP
Cryptocephalinae	*Cryptocephalus leonhardi* Breit	0	0	0	0	0	0	0	1	1	0	0	0	0	0	APPE
Cryptocephalinae	*Cryptocephalus lostianus* Burlini	0	0	0	0	0	0	0	0	0	0	0	1	1	0	SACO
Cryptocephalinae	*Cryptocephalus paganensis* Pic	0	0	0	0	0	0	0	1	0	0	0	0	0	0	APPC
Cryptocephalinae	*Cryptocephalus plantaris* Suffrian	0	0	0	0	0	0	0	0	0	0	1	0	0	0	SICI
Cryptocephalinae	*Cryptocephalus samniticus* Leonardi & Sassi	0	0	0	0	0	1	1	1	1	1	0	0	0	1	ITAL
Cryptocephalinae	*Cryptocephalus stragula* Rossi	0	0	0	0	1	1	1	1	1	1	0	0	0	0	ALAP
Cryptocephalinae	*Cryptocephalus tardus* Weise	0	0	0	1	0	0	0	0	0	0	0	0	0	0	ALPW
Cryptocephalinae	*Cryptocephalus zoiai* Sassi	0	0	0	0	1	0	0	0	0	0	0	0	0	0	ALSW
Cryptocephalinae	*Pachybrachis alpinus* Rapilly	0	0	0	0	1	0	0	0	0	0	0	0	0	0	ALSW
Cryptocephalinae	*Pachybrachis burlinii* Daccordi & Ruffo	0	0	0	0	0	0	0	0	0	0	0	0	0	1	TYRR
Cryptocephalinae	*Pachybrachis cinctus* Suffrian	0	0	0	0	0	0	0	0	0	0	0	1	1	0	SACO
Cryptocephalinae	*Pachybrachis fraudolentus* G. Müller	0	0	1	0	0	0	0	0	0	0	0	0	0	0	ALSE
Cryptocephalinae	*Pachybrachis osellai* Daccordi & Ruffo	0	0	0	0	0	0	0	0	0	0	1	0	0	0	SICI
Cryptocephalinae	*Pachybrachis ruffoi* Burlini	0	0	0	0	0	1	0	1	1	0	1	0	0	0	ITAL
Cryptocephalinae	*Pachybrachis salfii* Burlini	0	0	1	0	1	1	1	1	1	1	0	0	0	0	ALAP
Cryptocephalinae	*Pachybrachis sassii* Montagna	0	0	0	0	0	0	0	0	0	0	0	0	0	1	TYRR
Cryptocephalinae	*Pachybrachis siculus* Weise	0	0	0	0	0	0	0	0	0	0	1	0	0	0	SICI
Cryptocephalinae	*Pachybrachis testaceus* Perris	0	0	0	0	0	0	0	0	0	0	1	1	1	0	TYRR
Cryptocephalinae	*Stylosomus corsicus* Rey	0	0	0	0	0	0	0	0	0	0	0	1	1	0	SACO
Galerucinae	*Arima brachyptera* (Küster)	0	0	0	0	0	0	0	0	1	0	1	0	0	0	SISA
Galerucinae	*Arima buai* Havelka	0	0	0	0	1	0	0	0	0	0	0	0	0	0	ALSW
Galerucinae	*Arima maritima* Bua	0	0	0	0	1	0	1	0	0	0	0	0	0	0	AWNA
Galerucinae	*Calomicrus rottenbergi* Ragusa	0	0	0	0	0	0	0	0	0	0	1	0	0	0	SICI
Galerucinae	*Galeruca abbreviata* (Joannis)	0	0	0	1	1	0	0	0	0	0	0	0	0	0	ALPW
Galerucinae	*Galeruca corsica* (Joannis)	0	0	0	0	0	0	0	0	0	0	0	0	1	0	CORS
Galerucinae	*Galeruca nebrodensis* Ragusa	0	0	0	0	0	0	0	0	0	0	1	0	0	0	SICI
Galerucinae	*Galeruca reichei* (Joannis)	0	0	0	0	0	0	0	0	0	1	1	0	0	0	SISA
Galerucinae	*Galeruca sicana* (Reiche)	0	0	0	0	0	0	0	0	1	0	1	0	0	0	SISA
Galerucinae	*Luperus biraghii* Ragusa	0	0	0	0	0	0	0	1	1	1	1	0	0	0	APSI
Galerucinae	*Luperus calabricus* Laboissière	0	0	0	0	0	0	0	0	1	1	0	0	0	0	APPS
Galerucinae	*Luperus fiorii* Weise	0	0	0	0	0	0	0	1	0	0	0	0	0	0	APPC
Galerucinae	*Luperus leonardii* Fogato	0	0	1	1	1	1	1	1	1	1	0	0	0	0	ALAP
Galerucinae	*Luperus maculicornis* Desbrochers	0	0	0	0	0	0	0	0	0	0	0	0	1	0	CORS
Galerucinae	*Luperus pygmaeus* Joannis	0	0	0	0	0	1	1	1	1	1	0	0	0	1	ITAL
Galerucinae	*Luperus ragusai* Laboissière	0	0	0	0	0	0	0	0	1	0	1	0	0	0	SISA
Galerucinae	*Luperus revelierei* Perris	0	0	0	0	0	0	0	0	0	0	0	1	1	0	SACO
Galerucinae	*Luperus vitalei* Ragusa	0	0	0	0	0	0	0	0	0	0	1	0	0	0	SICI

**Figure 2.**
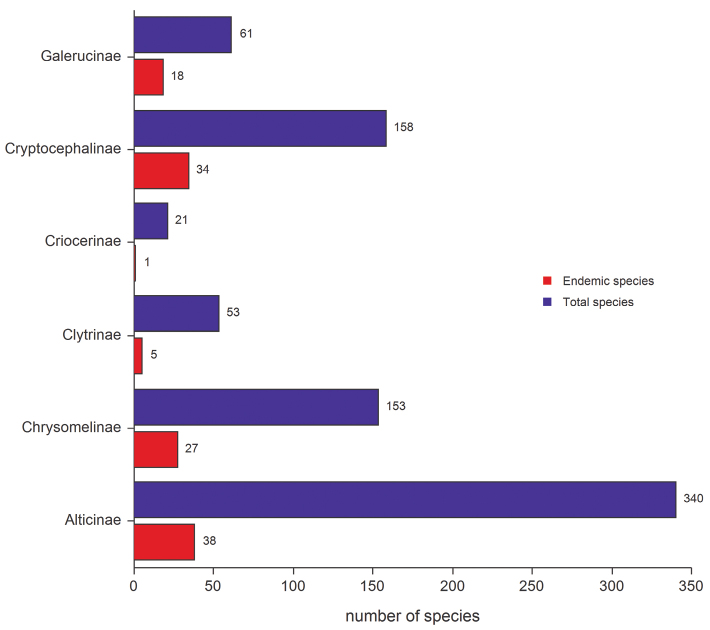
Total number of species and endemic species for the leaf beetle subfamilies occurring in the study area.

The percentage of endemic species is not proportionate to the different subfamilies of Chrysomelidae (Fig. 2). Alticinae, for example, display the greatest endemic species richness (38), but only represent 11.18% of the entire Italian flea beetle fauna (340 species). In some of the other subfamilies the percentage of endemic species is higher than the total average of 14.82% for the Chrysomelidae in Italy. Examples include: the Galerucinae with 29.51% (18 endemic species from a total of 61); Cryptocephalinae with 21.52% (34 endemic species from a total of 158); and Chrysomelinae with 17.65% (27 endemic species from a total of 153). Besides the Alticinae, lower endemic species percentages are also found in Clytrinae with 9.43% (5 endemic species from a total of 53) and Criocerinae with 4.76% (1 endemic species from a total of 21). No endemic species of Cassidinae, Donaciinae, Eumolpinae, Hispinae or Lamprosomatinae were found in the study area.

**Figure 3.**
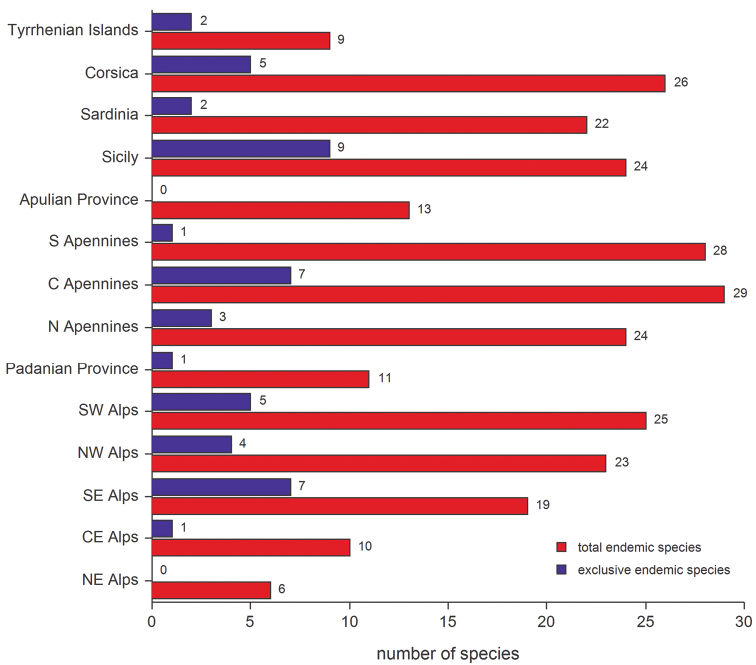
Number of endemic and exclusively endemic species occurring in all geographic sectors studied, depicting the: Apulian Province; South-Western, North-Western, South-Eastern, Central-Eastern and North-Eastern Alps; Central, Northern, and Southern Apennines; Corsica; Padanian Province; Sardinia; Sicily; and the small Tyrrhenian Islands.

Regions with the greatest richness of endemic leaf beetle species are (Fig. 3): the Central Apennines (29 species), Southern Apennines (28), Corsica (26), South-Western Alps (25), Northern Apennines (24), Sicily (24), North-Western Alps (23) and Sardinia (22). Areas with the poorest endemic species richness are: the North-Eastern Alps (6), Tyrrhenian Islands (9), Central-Eastern Alps (10), Padanian Province (11), and Apulian Province (13). However, if we only consider the local exclusively endemic species for every region, the greatest endemic species richness can be found in Sicily (9 species), the Central Apennines (7), and South-Eastern Alps (7) (Fig. 3). Local exclusively endemic species can provide important information about past and present isolation conditions for a given geographical region. At this point it is noteworthy that the number of exclusively endemic species for Corsica (5), and especially for Sardinia (2), is very low. However, if we consider the Sardinian-Corsican area as a whole, the number increases to 17 species, demonstrating the common history shared by these two islands and their intensive faunistic exchange. The Apulian Province and the North-Eastern Alps have no exclusively endemic species. The former probably because the Apulian Province was never isolated geographically or ecologically; the latter because the North-Eastern Alps, besides a poor endemic species richness, display ecological continuity that has possibly promoted horizontal range expansions into other adjacent Alpine sectors, thus hampering local endemization.

**Figure 4.**
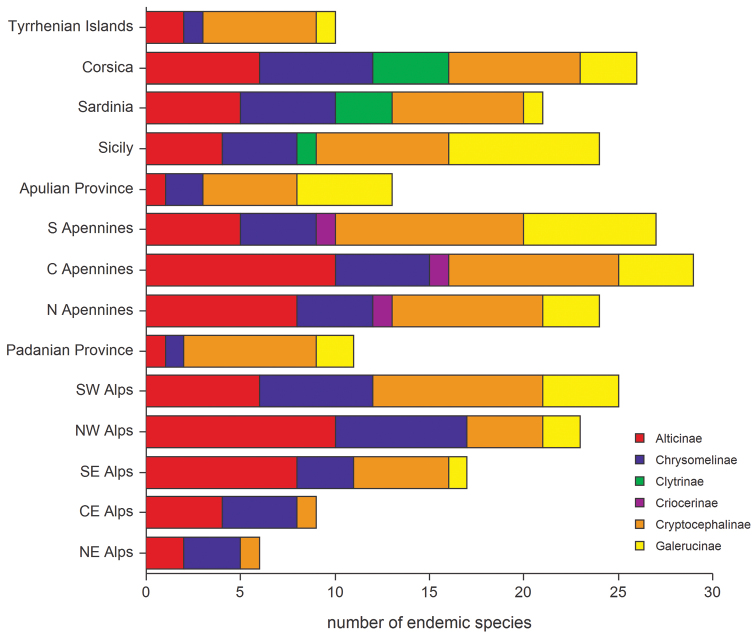
Number of endemic species for leaf beetle subfamilies occurring in the geographical sectors studied.

The endemic component of the different subfamilies for all the regions studied (Fig. 4) is, in general, significantly correlated with an increase in altitude or the measure of insularity; in the largest islands these two factors often fulfill a synergistic role. Regarding the altitude, the highest number of endemic species occur between 400 m and 1800 m a.s.l. with the majority found between 700 m and 800 m; the species from the plains have been added to the low and medium altitude species (Fig. 9). Endemization associated with lower altitudes generally occurs in insular areas, particularly Sardinia, Corsica and the small Tyrrhenian islands; whereas endemization associated with higher altitudes mainly occurs in the Central Apennines, where the alternation of catathermic and hypsothermic phases during the Pleistocene glaciations played an important role in the isolation and differentiation of the montane fauna. The number of endemic species and area are not significantly correlated (Fig. 8).

**Figure 5.**
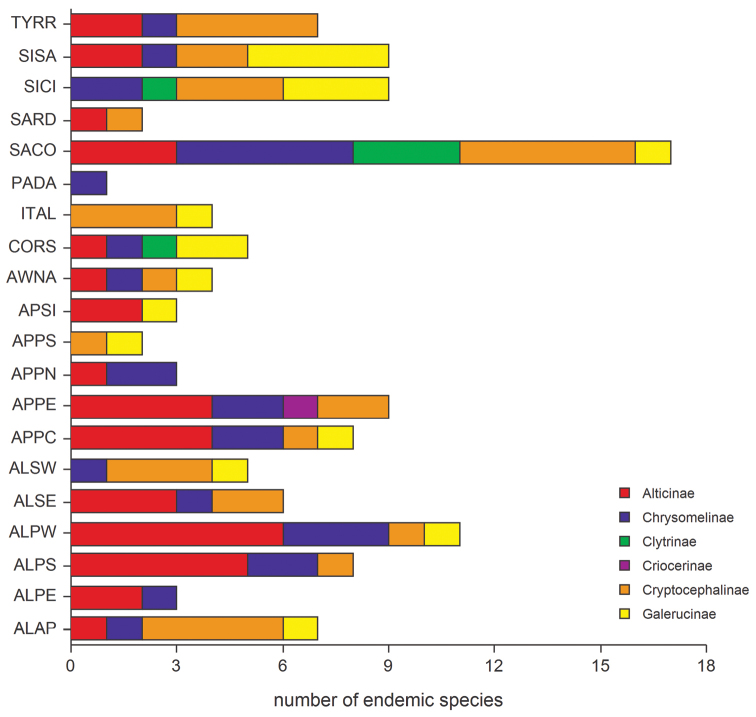
Number of endemic species in the different leaf beetle subfamilies, based on their distribution (for abbreviations see the text).

Based on the recognized distribution types for the Italian endemic fauna, as proposed by Vigna Taglianti et al. (1999) and Stoch and Vigna Taglianti (2006), and partially modified by us (see “Materials and methods” above), we can conclude that the Sardinian-Corsican type (SACO) in which Cryptocephalinae and Chrysomelinae are dominant, and the Western Alpine type (ALPW) where Alticinae are clearly in the majority, represent the distributions with the greatest abundance of endemic species. However, other distribution types that are well represented are the: Sicilian (SICI), where the absence of Alticinae has to be emphasized; Sicilian-Southern Apennine (SISA), where the Galerucinae are more abundant; Appenine (APPE) and Central Apennine (APPC), where Alticinae and, to a lesser extent, Chrysomelinae, Cryptocephalinae and Galerucinae, are more plentiful (Fig. 5). Distribution types with poor representation are the: Padanian (PADA), with *Chrysolina schatzmayri* (G. Müller) being the only species; and the Southern Apennines (APPS), with *Cryptocephalus daccordii* Biondi and *Luperus calabricus* Laboissière, being the only species - the latter also occurring in the Apulian Province (Fig. 5).

The results of the cluster analysis are depicted in a dendrogram (Fig. 10). In broad terms, it shows an “Alpine block” and an “Apennine-insular block” which are distinctly separated. The Alpine block is, in turn, subdivided into the Eastern Alps [(NE Alps - CE Alps) SE Alps] and Western Alps (NW Alps - SW Alps); whereas the Apennine-insular block shows a clear separation between the Apennines [(Apulian Province - S Apennines) C Apennines (Padanian Province - N Apennines)], and the small Tyrrhenian islands (Tyrrhenian Is.), Sicily (Sicily) and the Corsican-Sardinian region (Corsica-Sardinia). Within the Apennine block, the central and southern sectors (the Apulian Province included) show a higher faunistic similarity among them than with the northern sector (Padanian Province included). Finally, the position of Sicily reflects its close geographic proximity and ecological continuity with the Apennines.

**Figure 6.**
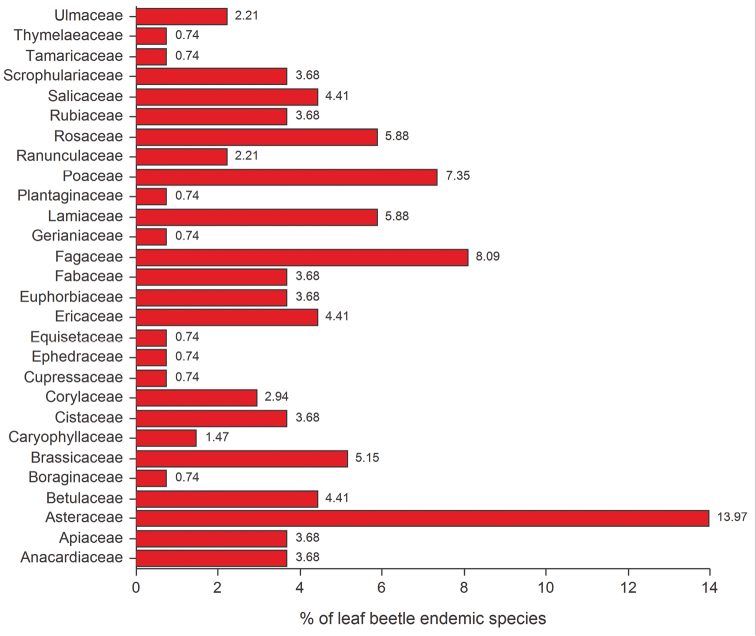
Percentage of endemic species associated with specific plant families.

**Figure 7.**
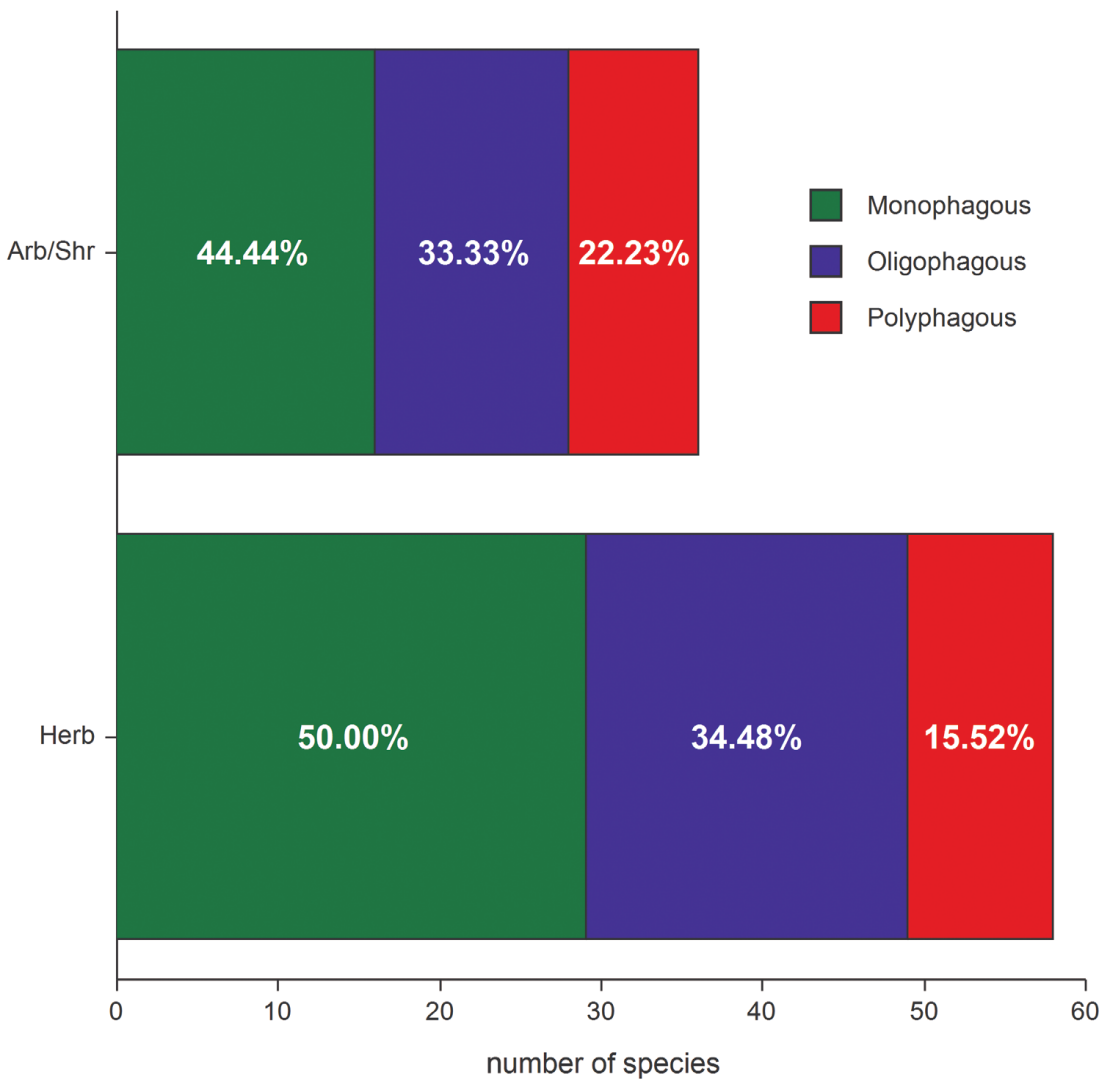
Percentage of endemic species within particular trophic categories.

Host plant families preferred by the endemic species occurring in the study region are, among herbaceous plants, the Asteraceae (13.97%), Poaceae (7.35%), Lamiaceae (5.88%) and Brassicaceae (5.15%); whereas among arboreal and shrubby plants, the Fagaceae (8.09%) and Rosaceae (5.88%) (Fig. 6) are dominant. The endemic leaf beetle species studied show an increase in trophic specialization. This is demonstrated by a high prevalence of monophagous (Herb: 50.00%; Arb/Shr: 44.44%) and, to a lesser extent, oligophagous elements (Herb: 34.48%; Arb/Shr: 33.33%), while species associated with herbaceous plants show the lowest percentage (15.52%) of polyphagous elements (Fig. 7).

The results of the parsimony analysis of endemicity (PAE) are reported in Fig. 11. This analysis reveals that the most significant region, displaying the richest endemicity, is the Alps:

- the CE Alps (Western Tauern and Eastern Tauern Alps) (quadrats C15, D13-14), are mainly characterized by sharing the endemic species *Neocrepidodera simplicipes* (Kutschera) and *Phyllotreta ziegleri* Lohse;
- the SE Alps (Julian Alps, Venetian Prealps, Dolomites, Carnic Alps) (E13-16, F11-12, F14), are characterized by the presence of *Aphthona juliana* Springer, *Neocrepidodera obirensis* (Ganglbauer), *Orestia carnica* Leonardi, *Orestia carniolica* Weise and *Pachybrachis fraudolentus* G. Müller;
- the Central Alps (Rhaetian and Bergamasque Alps) (E9, F7-9, G8), are mainly characterized by the presence of *Cryptocephalus barii* Burlini, but also by *Orestia heikertingeri* Leonardi;
- the NW Alps (Pennine, Cottian and Graian Alps) (F5, G4-6, H4, I3-4), are mainly characterized by the presence of *Cryptocephalus tardus* Weise, *Neocrepidodera adelinae* (Binaghi), *Neocrepidodera basalis* (K. Daniel), *Neocrepidodera nobilis* (J. Daniel), *Oreina canavesei* Bontems and *Pachybrachis alpinus* Rapilly, but also by *Orestia heikertingeri*; and
- the SW-Alps (Maritime and Ligurian Alps) (J4-5, K5), are characterized by the presence of *Arima buai* Havelka, *Cryptocephalus atrifrons* Abeille, *Cryptocephalus zoiai* Sassi and *Neocrepidodera ligurica* (J. Daniel).

In the remaining peninsular and insular regions, the following were also detected by the PAE (Fig. 11):

- many strictly local endemic species such as *Cryptocephalus paganensis* Pic, *Longitarsus springeri* Leonardi, *Luperus fiorii* Weise, *Oreina sibylla* (Binaghi), *Orestia brandstetteri* Kapp, *Psylliodes biondii* Leonardi, *Psylliodes springeri* Leonardi and *Timarcha fracassii* Meier occur in the Central Apennines (N14-16, O15-16);
- the exclusively endemic species *Luperus vitalei* Ragusa, *Calomicrus rottenbergi* Ragusa, *Galeruca nebrodensis* Ragusa and *Gonioctena theae* Baviera occur in Northern Sicily (Madonie and Nebrodi) (X16-17); and
- Western Corsica (N7, O7, P7) is characterized by the presence of *Galeruca corsica* (Joannis), *Labidostomis syriaca* Lacordaire, *Luperus maculicornis* Desbrochers, *Orestia coiffaiti* Doguet and *Timarcha cornuta* Bechyné.

Finally, in addition to the areas of endemism determined by the PAE in Fig. 11 we have also added the regions with restricted endemic species, represented by a single quadrat, namely: the Apuan Alps (J9) with *Timarcha apuana* Daccordi & Ruffo and *Chrysolina osellai* Daccordi & Ruffo; the Giglio Island (N10) with *Pachybrachis sassii* Montagna; the Pontine Islands (Q14) with *Pachybrachis burlinii* Daccordi & Ruffo; the Aegadian Islands (X13) with *Pachybrachis osellai* Daccordi & Ruffo; and finally Gennargentu (S8), with *Cryptocephalus alnicola* Costa.

**Figure 8.**
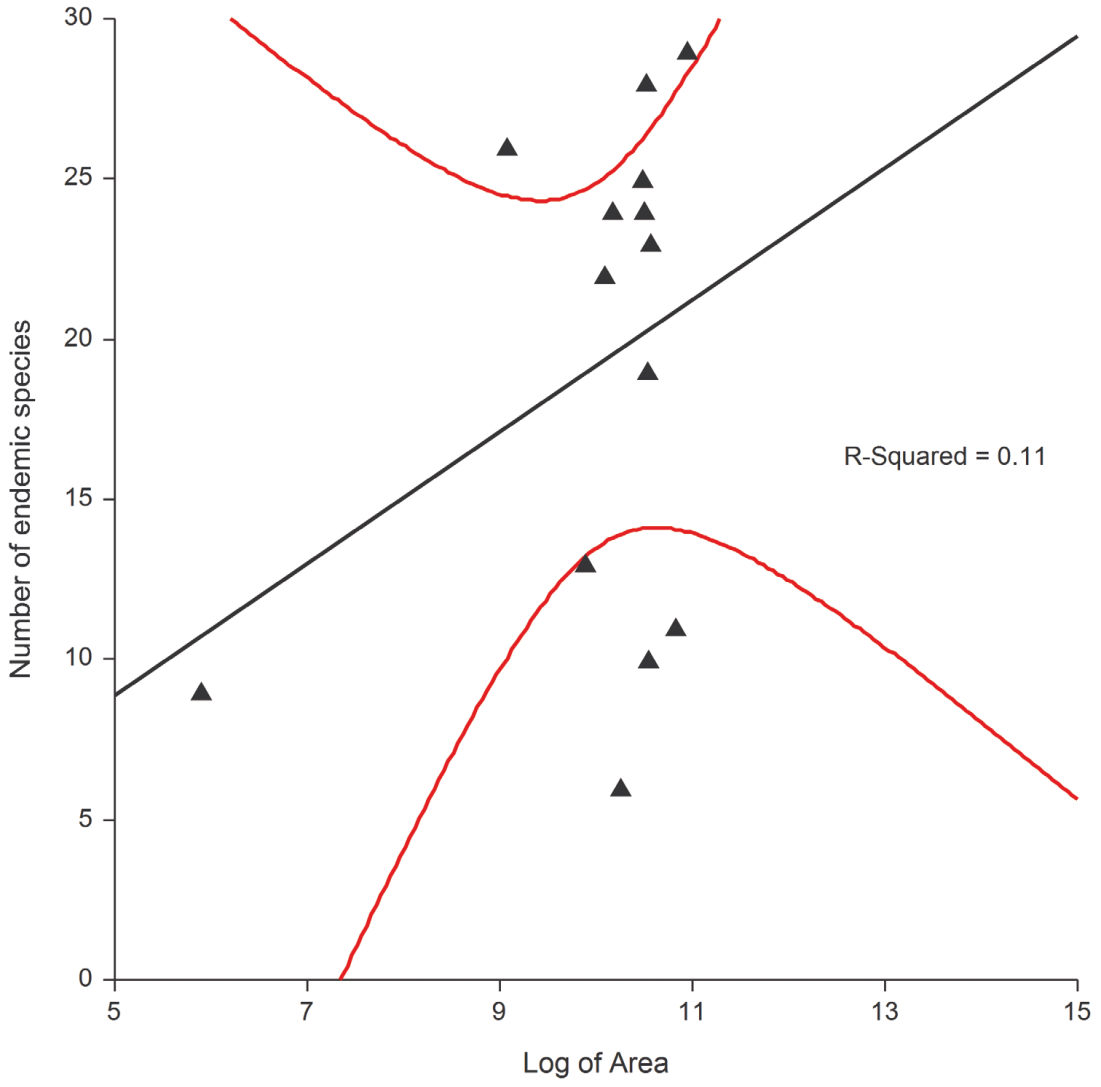
Number of endemic species to logarithm of area relationship.

**Figure 9.**
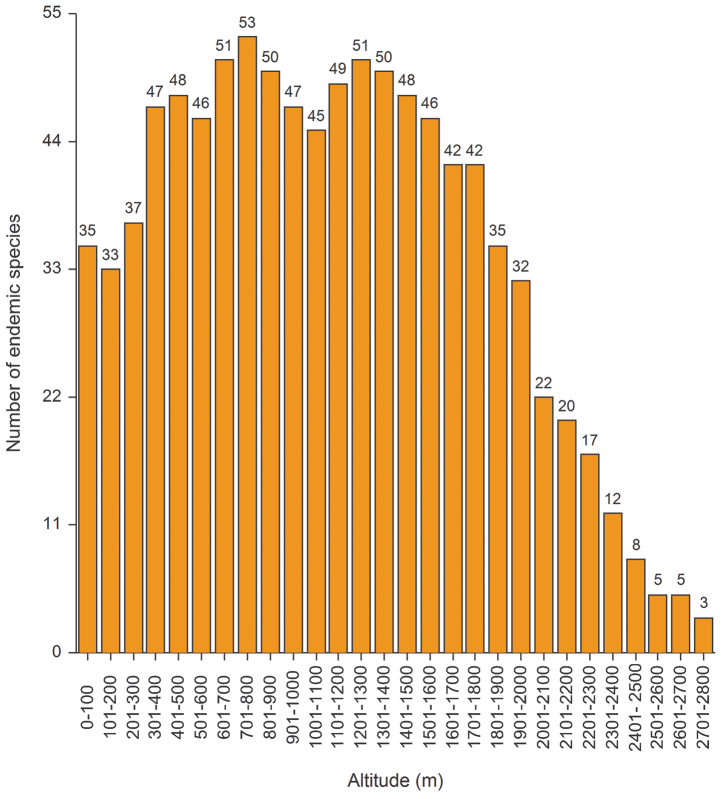
Number of endemic species associated with particular altitudinal intervals.

**Figure 10.**
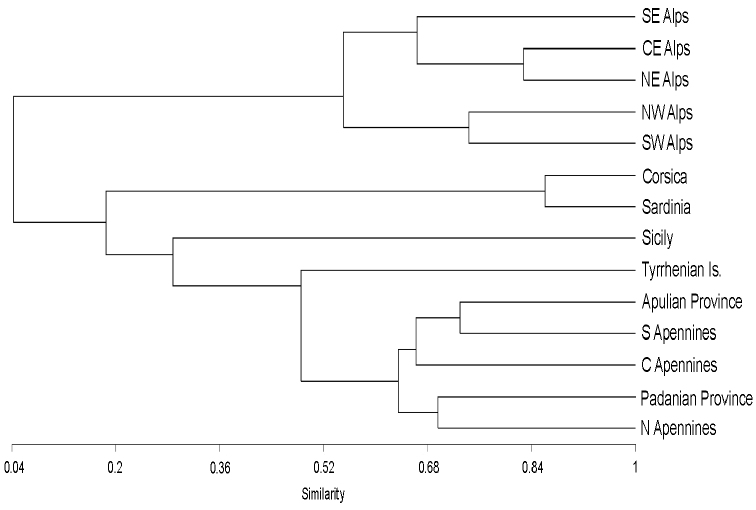
Dendrogram of endemic faunal similarity among the regions studied.

**Figure 11.**
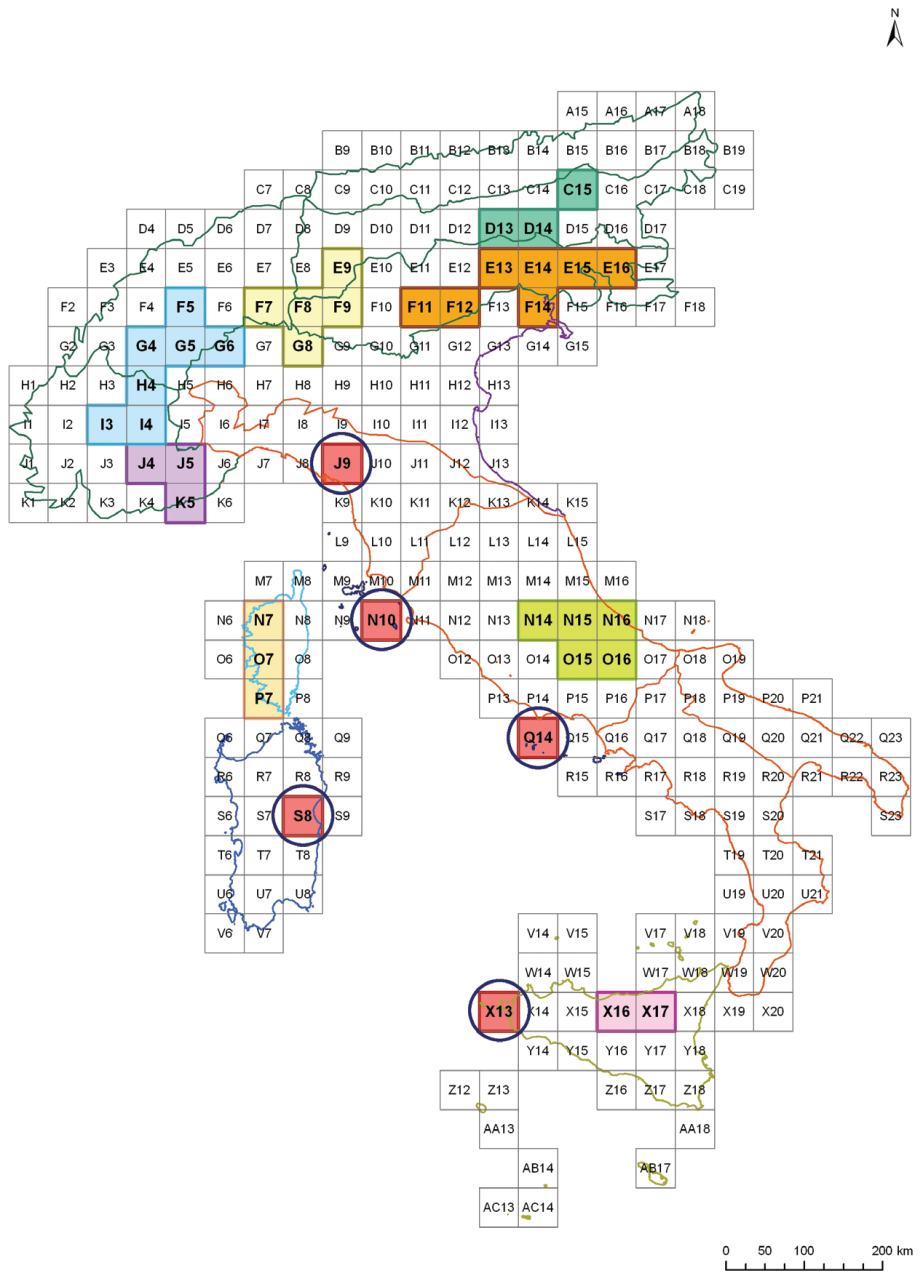
Areas of endemism identified by the parsimony analysis of endemicity (PAE) and single quadrats highlighting strictly local endemisms in red.

## Discussion

Chrysomelidae contribute significantly to the Italian endemic fauna, with 123 known endemic species representing 14.82% of the entire leaf beetle fauna for the country.

In the Alps, and particularly in the Apennines, the majority of endemic and subendemic species most likely originated as a result of the range shifts caused by the cyclic climatic changes during the Pleistocene glaciations. These climatic changes strongly influenced the recent biogeographic history of the faunas currently occurring in the high altitude montane systems of Europe (Triponez et al. 2011). Italy, in particular, represented one of the most important ice-age refugia for Mediterranean and Alpine species in Europe during the Pleistocene (Schmitt 2009; Taberlet et al. 1998). In the Alps the largest concentrations of strictly endemic species are in the south-eastern and western sectors, areas that were at lower altitudes and not covered by ice during glaciation (cf. Schmitt 2009). These ice-free areas in some sectors of the Alps, as also in the Central Apennines, most likely served as centres of glacial survival from where these species performed only altitudinal shift, but no major horizontal range expansions to other deglaciating areas. On the other hand, endemic species currently confined only to high alpine habitats in the inner Alps, such as *Neocrepidodera nobilis*, *Neocrepidodera obirensis*, *Neocrepidodera simplicipes*, *Neocrepidodera spectabilis* (J. Daniel), *Oreina peirolerii* (Bassi), *Oreina melancholica* (Heer), and *Cryptocephalus tardus*, may instead have survived *in situ* in the small ice-free areas topping the ice shield, the so-called “nunataks”, during the catathermic periods of the glaciations.

The Apennines have played an important role as a centre for differentiation and currently host 50 endemic species of leaf beetles, 30% of which are shared with the Alps, and 26% with Sicily. Some species, such as *Luperus fiorii*, *Oreina sibylla*, *Psylliodes biondii* and *Psylliodes springeri*, are faunistic elements of northern origin that found suitable ecological conditions for their survival at the highest altitudes of montane systems, such as Sibillini, Laga, Gran Sasso and Maiella, during the inter- and post-glacial hypsothermic periods. In other Apennine sectors, during the “climatic optimum” after the last glaciation, the increase in dominance of woody vegetation strongly reduced the presence of high altitude refugia, so causing the definitive disappearance of the cold-adapted species that had found refuge on isolated mountain massifs (cf. D’Alessandro and Biondi 2007; La Greca 2002).

Some endemic species of the Central and Southern Apennines, for example *Chrysolina sirentensis* (Meier), probably had a trans-adriatic origin instead (Bourdonné and Doguet 1991; Daccordi and Ruffo 1979, 1988, 2005; D’Alessandro and Biondi 2007). This distributional pattern is due to the effect of marine transgressions that occurred during the glacial phases, which allowed the formation of terrestrial bridges from the Balkan Peninsula and Central Italy and vice versa, particularly promoting the transit of submontane mesophilous elements (Gridelli 1950).

Other endemic Apennine species seem to belong to more ancient stock, generally pre-quaternary, that includes both Paleo-Mediterranean and possible Tertiary Alpine elements (Ruffo 1971). Among them are:

- *Longitarsus laureolae* Biondi, occurring in the Southern Apennines and North-Western Sicily. This species is very closely related to the West Mediterranean *Longitarsus candidulus* (Foudras), and *Luperus leonardii* Doguet endemic to the Pyrenees (Biondi 1988b);

- *Chrysolina osellai*, which is endemic to the Apuan Alps and belongs to a Paleo-Mediterranean species group with possible Pyrenean affinities (Daccordi and Ruffo 2005); and

- *Longitarsus springeri*, a species that is taxonomically distinct from the other congeneric European and Mediterranean entities (Leonardi 1975).

Among the species studied, only *Dibolia alpestris* Mohr shows a disjunct Alpine-Central Apennine distribution. Considering the absence of significant diagnostic phenotypic characters between these populations, it is possible to suppose that this species only reached the Central Apennines during the last glaciation.

The results from the PAE also show that the most important determining factor for the individualization of the areas of endemism, both in the Alps and in the Apennines, is the altitude. As reported above, this fact is surely due to historical events, and mainly the Pleistocene glaciations that promoted important differentiation phenomena as a consequence of relictual conditions, but also due to the subsequent role of high altitude montane environments in conserving and supporting animal populations that would otherwise have disappeared at lower altitude, because of human pressure.

Sicily has 14 subendemic species, the equivalent of 62.5% of the entire endemic leaf beetle component. Of these 13 are shared with the Southern Apennines and only one, *Pachybrachis testaceus* Perris which is of probable Paleo-Mediterranean origin, is shared with Sardinia and Corsica. The nine exclusively endemic species include some pre-Quaternary elements, such as *Timarcha sicelidis* Reiche which belongs to an ancient group (Miocene?) and possibly has affinities with *Timarcha cornuta*, endemic to Corsica, and *Timarcha sardea* Villa & Villa, endemic to Sardinia and Corsica (Daccordi and Pistarino 2001). No phylogeographical data are available for *Timarcha sicelidis* occurring in Northern Sicily and the Hyblean area, but its biogeographical history could most likely be analogous to the history of other taxa, such as the tenebrionid beetles *Pimelia grossa* Fabricius and *Pimelia rugulosa* Germar (Stroscio et al. 2011). The current distribution of these two species reflects the extensive geological changes over the Plio-Pleistocene period, that have deeply influenced the origin and distribution of the Sicilian fauna. Most of the exclusively endemic species, such as *Calomicrus rottenbergi*, *Galeruca sicana* (Reiche), *Galeruca nebrodensis*, *Gonioctena theae*, *Lachnaia caprai* Grassoand *Luperus vitalei*,are limited to Northern Sicily. Only *Cryptocephalus plantaris* Suffrian has a limited distribution in southern Sicily and Malta (Sassi and Zoia 2002); its origin can probably be traced back to marine regressions of the Pleistocene, when Malta and the Hyblean region were occasionally in contact (cf. Bonfiglio et al. 2002), or due to a more recent colonization of Sicily from the South.

The Sardinian-Corsican leaf beetle fauna comprises a great number of exclusively endemic species (24), including some pre-Quaternary elements of probable Miocene origin, such as *Timarcha cornuta* and *Timarcha sardea* (Daccordi and Pistarino 2001; Daccordi and Ruffo 1988).

Finally, among the possible Paleo-Mediterranean elements, we find the following: *Aphthona wagneri* Heikertinger, occurring in Corsica and on the small Tyrrhenian Islands (Tuscany Islands); *Aphthona perrisi* Allard and *Cryptocephalus biondii* Sassi & Regalin occurring in Sardinia, Corsica and on the Tuscany Islands; the above-mentioned *Pachybrachis testaceus*, occurring in Sardinia, Corsica, Sicily and on the circum-Sicilian islands.

In conclusion we can affirm that endemization phenomena in this beetle family seem mainly due to factors as philopatry, trophic specialization, meiopterism and adaptation to high altitudes, often in combination with vicariance and colonization events, which have contributed to create reproductively isolated units in the course of the time (cf. Piper and Compton 2010).

About endemization as consequence for adaptation to high altitudes, it can be due to historical events, mainly the Pleistocenic glaciations, that promoted important differentiation phenomena as consequence of relictuality conditions, but can also be due to the subsequent role of high montane environments in conservation, supporting animal populations that would otherwise have disappeared at lower altitude because of human pressure.
